# Development of the Attitudes Toward Partners of People Who Have Sexually Offended Questionnaire

**DOI:** 10.1007/s10508-025-03258-4

**Published:** 2025-12-17

**Authors:** Lea C. Kamitz, Theresa A. Gannon

**Affiliations:** 1 Centre for Research and Education in Forensic Psychology, School of Psychology, University of Kent, Canterbury, UK; 2 Present Address: International Policing and Public Protection Research Institute, Anglia Ruskin University, Chelmsford, CM1 1SQ UK

**Keywords:** Non-offending partners, Sexual offending, Attitudes, Sex offender

## Abstract

Non-offending partners of those who have sexually offended face severe negative consequences in the aftermath of an offense, many of which are directly linked to their negative interactions with others—including intervening agencies. Despite this, the only available measure of attitudes toward non-offending partners has several shortcomings and, as a result, attitudes toward non-offending partners are underexamined. The current research aimed to address this issue by using the input of Criminal Justice System-adjacent professionals, non-offending partners, and the general public to create a scale measuring Attitudes toward Partners of People who had Sexually Offended. Exploratory factor analysis revealed that attitudes toward non-offending partners had four underlying dimensions: (1) Judgement of non-offending partners’ relationship decision-making, (2) behavioral intent toward non-offending partners, (3) judgement of non-offending, and (4) shaming of non-offending partners. Subsequent studies validated the scale using confirmatory factor analysis, psychometric evaluations, and construct and criterion-related validity assessments. Here, we also found that professionals working for the police and social services held more favorable attitudes toward non-offending partners (i.e., score lower on the measure), than a general population sample, and that negative attitudes toward non-offending partners predicted intent to discriminate—but not behavioral aggression—toward this group. These findings are discussed, alongside the limitations of this research, in light of their implications and while considering avenues for future research.

## Introduction

Historically, research investigating the non-offending partners of individuals who have sexually offended—hereafter referred to as non-offending partners—has disproportionately focused on their usefulness for supporting and benefitting others. Here, non-offending partners have been conceptualized as being of benefit to the desistance of their partner who has offended by providing a stable relationship (McAlinden et al., 2017), as promoted in prominent rehabilitation frameworks (e.g., the risk-need-responsivity model; Bonta & Andrews, 2017). An exaggerated focus on the non-offending partner’s role after an offense has occurred is especially apparent in the opinion-based research on non-offending mothers (Tamraz, 1997). Here, non-offending partners have traditionally been used to safeguard children (Duff et al., 2017) or have even been blamed for the offenses committed by their partner. Surprisingly, the impact of the offense and its aftermath on non-offending partners themselves has so far been largely overlooked.

Nevertheless, recent streams of research (e.g., Armitage et al., 2024; Duncan et al., 2022; Kamitz & Gannon, 2024a; Kavanagh, 2022) have begun to investigate non-offending partners’ own experiences and to acknowledge their victim status as a result of their partners’ offending behavior. Here, it has emerged that non-offending partners commonly experience negative impacts due to their association with a stigmatized person, a process coined “courtesy stigma” (Goffmann, 1963; see also Condry, 2011). These negative impacts are practical, emotional, and social in nature, and are similar to those experienced by direct victims of crime (Brown, 2018; Duncan et al., 2020; Liddell & Taylor, 2015). Because of this, previous research that has acknowledged non-offending partners as victims has emphasized the complexity of non-offending partners’ support needs and the arising vulnerability of this population to stigmatization by groups that could provide such support. For example, in previous research, non-offending partners disclosed inconsistent, judgmental, or even hostile interactions with intervening agencies (e.g., police, social services, probation) in the aftermath of an offense (Armitage et al., 2024; Duncan et al., 2022; Kamitz & Gannon, 2024a). Such negative interactions, rather than providing the required support, can instead exacerbate trauma and courtesy stigma.

Research examining agency interactions with non-offending partners has mainly focused on how non-offending partners think they are perceived, rather than how they are actually perceived by others. Here, the research examining others’ attitudes toward non-offending partners has been extremely limited. Aside from the very specific focus on mother-blaming in cases of intrafamilial sexual abuse (for a review, see Azzopardi et al., 2018), to our knowledge, only one peer-reviewed article measuring attitudes toward non-offending partners has been published to date (Plogher et al., 2016). What is more, research has completely failed to investigate the attitudes toward non-offending partners held by Criminal Justice System-adjacent professionals. This is concerning given that non-offending partners commonly encounter such professionals and such professionals’ attitudes and reactions toward them may have an especially powerful impact on their well-being after an offense is disclosed (e.g., Kamitz & Gannon, 2024a).

While the previously mentioned research by Plogher et al. (2016) made an important contribution by developing a scale to measure attitudes toward non-offending partners and demonstrating that higher scores on this measure predict greater intent to discriminate against this population, several limitations warrant consideration when evaluating its broader applicability. First, the scale was developed within the specific cultural and legal context of the USA, where registration laws for individuals who have sexually offended differ significantly from those of other countries. These differences, such as the public availability of the US registry (The United States Department of Justice, n.d.) compared to more restricted access in countries like the UK (Beard, 2023), or a complete lack of registration in countries such as Germany (Thomas, 2011), may affect perceptions of the severity of offenses and, consequently, influence responses to scale items. While the scale references registration conditions in its preamble rather than explicitly embedding them within individual item wording, such differences may still limit its relevance to other cultural contexts.

Second, item development did not involve consultation with non-offending partners or professionals working with them. Instead, Plogher et al. (2016) developed items after consulting a small general population sample (*N* = 166). While this provides insight into broader societal perceptions, it overlooks the crucial perspectives of both non-offending partners and professionals in Criminal Justice System-adjacent agencies, who are key stakeholders in understanding and addressing this populations’ experiences. Incorporating such perspectives of the target population would significantly enhance the content validity of attitudinal measures (Boateng et al., 2018).

Third, the scale was administered exclusively to a general population sample of only 168 participants, which is unusually small for factor analysis. Best practices in psychometric research recommend a minimum of 10 participants per item (Nunnally, 1978; Siddiqui, 2013) or at least 200 participants (Comrey, 1988; Comrey & Lee, 1992; Guadagnoli & Velicer, 1988) for reliable factor analysis. Given the scale’s 51 items, the small sample size may have produced unstable factor loadings and limited generalizability (see Boateng et al., 2018). A larger sample size and confirmatory factor analysis would be required to robustly establish the scale’s factor structure (Guadagnoli & Velicer, 1988).

Lastly, Plogher et al. (2016) referred to non-offending partners as “partners of sex offenders,” a label that has been criticized for evoking stereotypical and stigmatizing connotations (Willis, 2018). Research has demonstrated that using such labels can prime more negative attitudes (Lowe & Willis, 2021) and decrease engagement with those who have sexually offended, specifically (Lowe & Willis, 2020). Given the stigma already faced by non-offending partners as a result of their association with someone who has sexually offended, adopting person-centered, non-stigmatizing language is essential for future measures to avoid reinforcing harmful stereotypes.

### The Current Research

To address and counteract potentially negative attitudes toward non-offending partners held by those who may encounter this population in a professional or personal capacity, and subsequently improve support and reduce traumatization, the nature of such negative attitudes, as well as their prevalence and magnitude first need to be examined. Due to the dearth of research in this field and the lack of a suitable scale assessing such attitudes, there were five aims to this multi-study research:

1. To develop a new measure of attitudes toward non-offending partners, using non-stigmatizing language and investigate the measure’s factorial structure (Pilot Study and Study 1);
2. To validate the arising factorial structure with an independent sample (Study 2);
3. To explore professionals’ scores on the new measure, and thus their attitudes toward non-offending partners, and compare these to attitudes held by a general population sample (Study 3);
4. To assess the measure’s construct and criterion-related validity by examining its associations with relevant variables and its ability to predict intent to discriminate against or support non-offending partners (Study 4), and finally;
5. To enhance the ecological validity of the previous validation study through a laboratory-based study investigating whether higher scores on the scale predict behavioral aggression toward non-offending partners (Study 5).

## Study 1: Scale Development

Prior to this study, we developed items during a pilot study for which we drew on the views of professionals working non-offending partners and insights from previous studies with non-offending partners and the general public. We then asked for experts’ opinions regarding the clarity and acceptability of the resulting items. The full pilot study can be found in the supplementary materials. In this study, the 50 items obtained from the pilot study stage were arranged into the Attitudes Toward Partners of People Who Have Sexually Offended (APPSO) measure so that item reliability, construct validity, and internal reliability could be examined.

### Method

#### Participants

We recruited at least 10 participants per item for our exploratory factor analysis (see Nunnally, 1978; Siddiqui, 2013 for recommendations). The final sample comprised 503 Prolific users (female: 50.3%, male: 48.9%, other: 0.6%, prefer not to say: 0.2%) who resided in the UK. Participants’ ages ranged between 18 and 79 (*M* = 41.08; SD = 14.24). Most participants were white (*n* = 440, 87.5%), while 6% (*n* = 30) were Asian, 4% (*n* = 20) were Black, African, or Caribbean, and 2% (*n* = 10) were from two or more ethnic groups. Four participants (0.8%) preferred not to indicate their ethnic group, and one participant (0.2%) indicated that they belonged to an ethnic group not listed.

We used Prolific throughout the online-based studies within this research due to its advantages over other crowd-sourcing platforms, such as Amazon MTurk (Palan & Schitter, 2018; Prolific, 2024). These include, but are not limited to, a more rigorous protocol of monitoring accounts for potential bot activity (Bradley, 2018), and overall higher data quality (Peer et al., 2022).

#### Measures

The study was advertised on Prolific on 2 November 2022 as “A Scale Measuring Attitudes Toward Non-Offending Partners of Individuals who Have Sexually Offended.” The questionnaire was administered using Qualtrics. After providing demographic information, participants were informed that “the following series of statements will assess your attitudes toward non-offending partners of people who have committed sexual offenses. Non-offending partners are not themselves involved in the offending and are also not a victim of their partner.” Then, participants were presented with the 50 items previously constructed during the pilot study, in randomized order, and asked to “indicate the degree to which you agree with each item. Be as honest as possible, there are no right or wrong answers.” Here, participants responded using a 7-point Likert scale (1 = *Fully disagree*, 7 = *Fully agree*), as recommended for bipolar items (Krosnick & Presser, 2010). Higher scores indicated more negative attitudes toward non-offending partners. Attention checks, of which participants were made aware prior to participation, were conducted throughout the survey. Participants failing two or more of the three attention checks (*n* = 19) were excluded from the analysis, in line with Prolific’s attention check policy (Prolific, 2022). Participants received £0.70 for their participation.

### Results

IBM SPSS Statistics 28 (IBM Corp., 2021) was used for the majority of analyses, while IBM SPSS Amos 28 (Arbuckle, 2021) was used to compute fit indices and correlations. The internal consistency coefficient *α* was interpreted according to Ponterotto and Ruckdeschel’s (2007) guidelines and Pearson’s zero-order correlation coefficient *r* was interpreted according to Funder and Ozer’s (2019) guidelines. Model fit was interpreted using Hu and Bentler’s (1999) cut-off values for fit indices: Root Mean Square Error of Approximation (RMSEA) ≥ .05; Comparative Fit Index (CFI) ≥ .95; Tucker–Lewis Index (TLI) ≥ .95.

#### Item Reliability

First, item discrimination indices were computed for each item using the extreme groups method comparing the lowest and highest scoring 27% of the population (Anastasi, 1982). Only items with good discrimination indices (≥ .30; McGahee & Ball, 2009) were retained resulting in 16 discarded items at this stage. Subsequently, we computed inter-item and item-total correlations. Here, 9 items with inter-item correlations below .15 (Clark & Watson, 1995) and 3 items with item-total correlations below .50 (Paulsen & BrckaLorenz, 2017) were examined and considered for deletion. Given the conceptual significance of most items, we chose to delete only two items since they exhibited both poor inter-item and poor item-total correlations.

#### Construct Validity and Internal Reliability

The Kaiser–Meyer–Olkin measure of sampling adequacy (KMO = .96, Kaiser, 1974) and Bartlett’s test of sphericity [χ^2^(496) = 11,795.87, *p* < .001; Bartlett, 1951] indicated that the data were suitable for exploratory factor analysis. Subsequently, exploratory factor analysis was conducted on the 32 remaining items. An examination of the scree plot (Cattell, 1966) in combination with the eigenvalues using the Kaiser rule (Kaiser, 1960)^1^ suggested the retention of four factors. The only item with a factor loading below .40 was removed at this stage (Raykov & Marcoulides, 2010). The final factorial solution obtained after extracting four factors using the maximum likelihood estimation explained 64.74% of the pre-rotation variance of the measure. As resulting factors were assumed to be correlated, an oblique rotation (Promax with Kaiser normalization) was performed. All remaining variables loaded significantly on at least one factor. As factor patterns above .30 could be considered significant at this sample size (Hair et al., 2009), three items were interpreted to cross-load on two factors. Given that it was conceptually sensical for these items to cross-load onto both factors, they were retained. After accounting for covariances between error terms of items loading onto the same factor (Collier, 2020), the model achieved a good fit according to the Root Mean Square Error of Approximation (RMSEA = .052, 90% CI = .047–.056, *p* = .290) and the Comparative Fit Index (CFI = .957). While the Tucker–Lewis Index was slightly low (TLI = .945), it was still within the range considered acceptable by most guidelines (e.g., Bentler & Bonnett, 1980). Table 1 shows the factor pattern and structure coefficients after rotation, as well as mean scores and standard deviations for each item. The chi-square statistic indicated poor model fit [χ^2^(360) = 839.81, *p* < .001]. However, this is to be expected as the chi-square test assumes multivariate normality (McIntosh, 2007) and nearly always rejects models when the sample size is large (Bentler & Bonnett, 1980).

**Table 1:** Study 1 exploratory factor analysis four-factor solution (*N* = 503)

APPSO item	Factor loading				*M* (SD)
	1	2	3	4	
*Factor 1: Judgement of relationship decision*					
1. Non-offending partners who stay in their relationship with a person who has sexually offended are prioritizing their relationship over their children's well-being and safety	**.86**	.001	.04	−.08	5.04 (1.59)
2. I don't understand how someone could possibly choose to stay in a relationship with a person who has sexually offended	**.85**	.06	−.16	−.03	5.16 (1.66)
3. Non-offending partners who stay in their relationship with someone who has committed a sexual offense are not doing enough to protect their children	**.84**	.12	.10	−.22	4.82 (1.59)
4. Non-offending partners who have children are putting them at risk by staying in their relationship with someone who has sexually offended	**.83**	−.02	−.08	−.05	5.40 (1.43)
5. There is no good reason for a non-offending partner to stay in their relationship after finding out that their partner has committed a sexual offense	**.76**	.05	−.03	.07	4.48 (1.70)
6. Non-offending partners are naive for staying with someone who has sexually offended	**.75**	−.12	.14	.09	4.59 (1.62)
7. Non-offending partners are disrespecting the victim by staying in the relationship with someone who has sexually offended	**.75**	−.02	.05	.08	4.35 (1.71)
8. Non-offending partners who stay in a relationship with someone who has sexually offended are hypocrites	**.73**	.01	−.05	.13	4.33 (1.71)
9. Non-offending partners are stupid for staying in their relationship after finding out about a partner’s sexual offending	**.72**	−.07	.10	.11	4.59 (1.63)
10. Non-offending partners who stay with someone who has sexually offended are putting themselves at risk	**.69**	−.11	.03	.03	5.27 (1.37)
11. Non-offending partners who stay with someone who has sexually offended do not care about their children	**.59**	.19	.24	−.14	3.69 (1.67)
12. Non-offending partners might have a good reason for continuing their relationship with someone who has sexually offended. (r)	**.58**	.06	−.08	.06	3.95 (1.57)
13. Non-offending partners are only worthy of support if they did not know about a partner’s sexual offense	**.55**	.06	−.01	−.01	4.49 (1.77)
14. Non-offending partners are in denial about their partners’ sexual offending	**.46**	−.09	.29	.15	4.29 (1.38)
*Factor 2: Behavioral intent*					
15. I would be understanding toward a non-offending partner of someone who has committed a sexual offense. (r)	.01	**.94**	−.10	−.06	3.25 (1.47)
16. I would be empathetic towards a non-offending partner of someone who has sexually offended. (r)	.03	**.83**	−.12	.03	3.15 (1.43)
17. Non-offending partners of those who have sexually offended deserve to be treated with respect. (r)	−.001	**.79**	−.10	.02	2.79 (1.38)
18. I would distance myself from a friend if I found out that they were in a relationship with someone who has sexually offended	.22	**.71**	−.13	.003	3.98 (1.79)
19. I would judge someone who is in a relationship with a person who has sexually offended	.19	**.64**	−.07	.001	4.21 (1.67)
20. Non-offending partners of people who have sexually offended deserve to be socially ostracized	−.13	**.61**	.18	.04	2.16 (1.3)
21. Non-offending partners of individuals who have sexually offended are immoral	−.08	**.57**	**.31**	.09	2.6 (1.37)
22. Non-offending partners of those who have committed sexual offenses are bad people	−.21	**.54**	**.44**	−.03	2.32 (1.35)
*Factor 3: Judgement of character*					
23. Non-offending partners of people who have sexually offended have a lower IQ than other people	.01	−.06	**.83**	−.07	2.84 (1.41)
24. Non-offending partners who stay in their relationship with a person who has sexually offended are mentally ill	.16	−.04	**.77**	−.15	3.02 (1.43)
25. Non-offending partners of individuals who have committed sexual offenses are timid and afraid	.13	−.12	**.73**	−.03	3.65 (1.35)
26. Non-offending partners of people who have sexually offended are weak(- minded)	.04	.09	**.60**	.19	3.29 (1.49)
27. Non-offending partners who claim they did not know about their partner’s sexual offending are lying	−.15	.19	**.50**	.11	2.69 (1.28)
28. Non-offending partners do not care about the victim of their partner’s sexual offending	−.05	**.36**	**.47**	.08	3.05 (1.45)
29. Non-offending partners of people who have committed sexual offenses are bad parents	.11	.23	**.44**	.04	3.23 (1.50)
*Factor 4: Shame*					
30. Non-offending partners of those who have sexually offended should feel ashamed	.03	.18	−.02	**.81**	3.25 (1.68)
31. Non-offending partners of people who have committed sexual offenses should feel embarrassed	.06	0.11	−.004	**.80**	3.51 (1.65)

Estimator = MLE, rotation = promax. Factor loadings ≥ .40 are in bold. CFI = .95; RMSEA = .05, 90% CI = 0.47–0.55, *p* = .33; TLI = 945. Higher scores = more negative attitudes. (r) = reverse-coded

The first factor comprised 14 items, which are presented in decreasing order of loadings (see Table 1 for all four factors). It had an eigenvalue of 13.79 and explained the highest amount of scale variance (44.48%). Inductive content analysis of the items loading onto this factor revealed that the 12 highest-loading items referred to non-offending partners’ relationship decision-making, specifically, whether the participant negatively judges a non-offending partner for choosing to stay in a romantic relationship with someone who has sexually offended (e.g., “I don’t understand how someone could possibly choose to stay in a relationship with a person who has sexually offended”). Often, this contained judgements of non-offending partners as stupid or naïve, or as being a bad parent for staying in the relationship. Only one item containing the word “stay” loaded significantly onto any other factor (Item 24). Thus, this factor was named Judgement of Relationship Decision. Its internal consistency was excellent (*α* = .95).

The second factor comprised 8 items. It had an eigenvalue of 3.76 and explained the second-highest amount of scale variance (12.13%). Content analysis revealed that items loading onto this factor primarily assessed how participants aimed to behave toward a non-offending partner (e.g., “I would be understanding toward a non-offending partner of someone who has committed a sexual offense”). Thus, this factor was named Behavioral Intent. The factor’s internal consistency was excellent (*α* = .92; incl. all cross-loading items).

The third factor, which comprised 7 items had an eigenvalue of 1.53 and explained the second-lowest amount of scale variance (4.93%). Content analysis showed that items loading onto this factor contained judgements about non-offending partners’ inherent characteristics, such as being stupid, vulnerable, or deceitful, irrespective of their relationship decision (e.g., “Non-offending partners of people who have sexually offended have a lower IQ than other people”). Thus, this factor was named Judgement of Character. Its internal consistency was excellent (*α* = .90; incl. all cross-loading items).

The fourth and final factor, which comprised two items had an eigenvalue of 0.99 and explained the lowest amount of variance (3.2%). This was retained due to its theoretical relevance and contribution to improved model fit, despite an eigenvalue of just below 1.00. Content analysis revealed that items loading onto this factor assessed which to degree participants believed non-offending partners should feel ashamed or embarrassed (e.g., “Non-offending partners of those who have sexually offended should feel ashamed”). Thus, this factor was named Shame. The factor’s internal consistency was excellent (*α* = .93). While the fourth factor only had two items, we chose to retain a four-factor solution as this markedly improved the model fit when compared to a three-factor solution.

Three Items (Items 21, 22 and 28) cross-loaded significantly onto Behavioral Intent (Factor 2) and Judgement of Character (Factor 3)*.* This may offer insight into a potential link between these two concepts, that is, that participants may indicate negative behavioral intent, such as wanting to socially distance themselves from non-offending partners, because they believe non-offending partners to have poor character. This may especially be the case as these items specifically assess poor character as being represented as immorality (e.g., “Non-offending partners of individuals who have sexually offended are immoral”), while other items assessing character primarily focus on poor character as represented by vulnerability (e.g., “Non-offending partners of individuals who have sexually offended are weak[-minded]”).

As expected, all extracted factors were significantly positively correlated, at *p* < .001. Each of these correlations was very large. Judgement of Relationship Decision was positively correlated with Judgement of Character (*r* = .72), Behavioral Intent (*r* = .50) and Shame (*r* = .60). Behavioral Intent was positively correlated with both Judgement of Character (*r* = .73) and Shame (*r* = .76). Lastly, Judgement of Character and Shame were also positively correlated (*r* = .76).

## Study 2: Scale Structure Validation

In this second study, the APPSO measure created in Study 1 was administered to a general population sample and confirmatory factor analysis conducted to assess whether the four-factor structure observed was reliable enough to fit data gathered from an independent sample.

### Method

#### Participants

We again aimed to recruit 10 participants per item (Nunnally, 1978; Siddiqui, 2013) resulting in a final sample of 308 Prolific users (female: 49.4%, male: 49%, other: 0.6%, prefer not to say: 1%) who resided in the UK. Participants’ ages ranged between 18 and 79 (*M* = 39.84; SD = 13.21). Most participants were white (*n* = 263, 85.4%), while 7.1% (*n* = 22) were Asian, 2.9% (*n* = 9) were from two or more ethnic groups, and 2.6% (*n* = 8) were Black, African, or Caribbean. Five participants (1.6%) indicated that they belonged to an ethnic group not listed, and 2 participants (0.6%) preferred not to indicate their ethnic group.

#### Measures

The study was advertised on Prolific on 21 November 2022 as “A Scale Measuring Attitudes Toward Non-Offending Partner of Individuals who Have Sexually Offended. Participants who had taken part in Study 1 were automatically excluded from participating. The procedure mirrored that of Study 1, with the exception that participants were only presented with the 31-item measure, instead of all items. Participants failing two or more of the three attention checks (*n* = 11) were again excluded from analysis, in line with Prolific’s attention check policy (Prolific, 2022). Participants received £0.50 for their participation.

### Results

All analyses were conducted using IBM SPSS Amos 28 (Arbuckle, 2021). Internal consistency coefficient *α* was interpreted according to Ponterotto and Ruckdeschel’s (2007) guidelines and Pearson’s zero-order correlation coefficient *r* was interpreted according to Funder and Ozer’s (2019) guidelines. Model fit was interpreted using Hu and Bentler’s (1999) cut-off values for fit indices: Root Mean Square Error of Approximation (RMSEA) ≥ .05 and Comparative Fit Index (CFI) ≥ .95; Tucker–Lewis Index (TLI) ≥ .95.

We conducted a confirmatory factor analysis to test the oblique four-factor solution that arose from exploratory factor analysis. First, measured variables were screened for non-normality and multivariate outliers. As no kurtosis value of any item was larger than 7, data were interpreted to adhere to univariate normality according to Byrne’s (2016) guidelines. However, a large multivariate kurtosis value suggested that multivariate normality was not achieved, and an examination of squared Mahalanobis distance values indicated the presence of multivariate outliers (Byrne, 2016). There were no missing data.

**Fig. 1:**
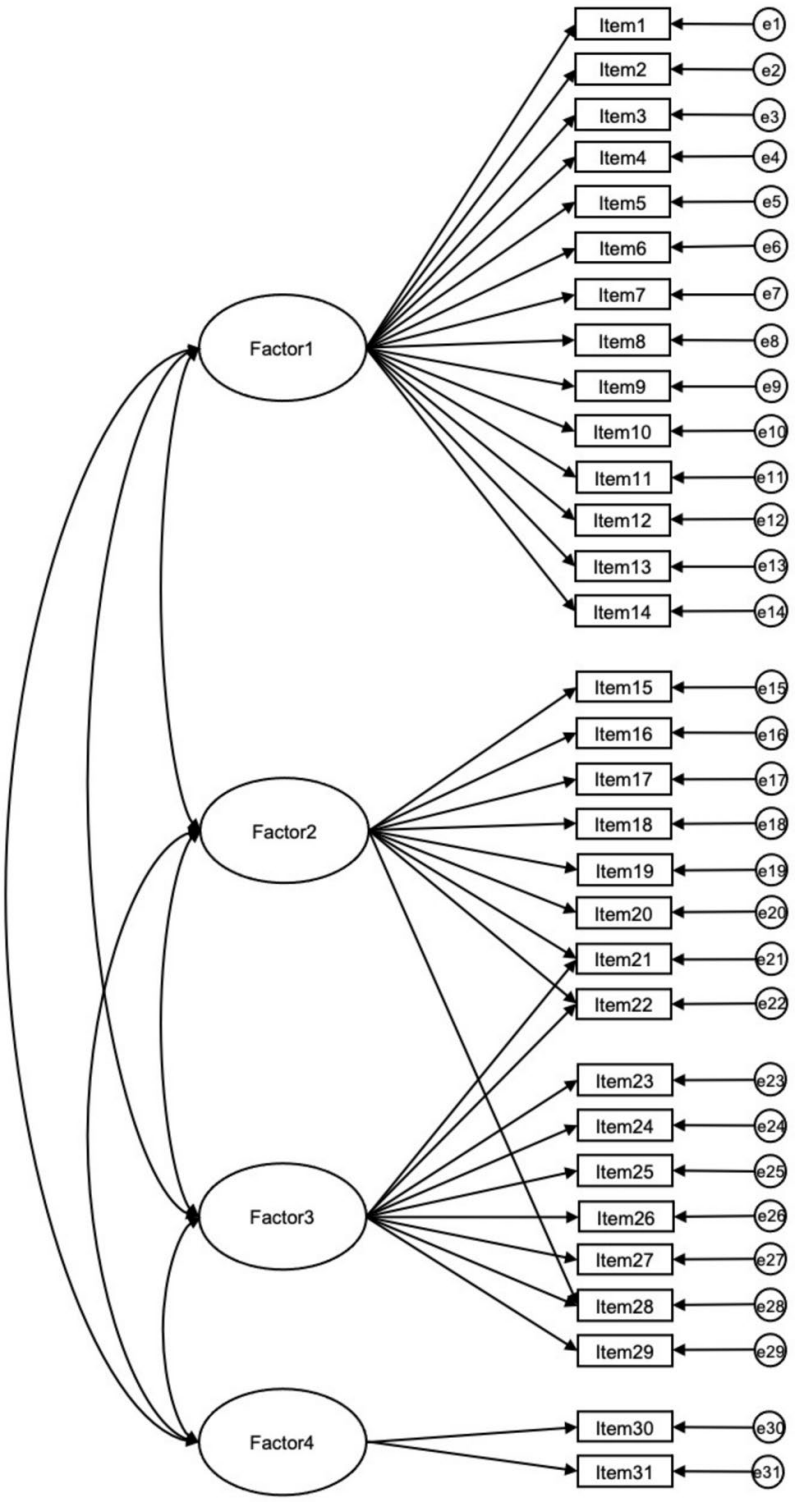
Oblique four-factor solution with maximum likelihood estimation. *Note.* Covariances between error terms are not represented visually but were accounted for in analysis

To reduce potential bias on standard error arising from multivariate non-normality, we conducted a maximum likelihood estimation with bootstrapping (1000 samples at 95% bias-corrected confidence level). Figure 1 shows the attempted model.

After accounting for covariances between error terms of items loading onto the same factor (Collier, 2020), the model achieved a good fit according to the Root Mean Square Error of Approximation (RMSEA = .053, 90% CI = .047–.060, *p* = .17) and the Comparative Fit Index (CFI = .955). While the Tucker–Lewis Index was slightly low (TLI = .942), it was still within the range considered acceptable by most guidelines (e.g., Bentler & Bonnett, 1980). The Chi-square statistic indicated poor model fit [χ^2^(361) = 676.57, *p* < .001]. However, as previously noted, this was to be expected as the Chi-square test assumes multivariate normality (McIntosh, 2007) and nearly always rejects models when the sample size is large (Bentler & Bonnett, 1980).

An examination of the regression weights demonstrated that almost all items loaded significantly onto the four factors as hypothesized. However, contrary to the model we observed during Exploratory Factor Analysis, Item 28 only loaded onto Factor 3 and did not cross-load onto Factor 2. This could possibly be the case as the judgement of non-offending partners as immoral, which we previously hypothesized to be a potential reason for the cross-loadings and a link between its latent constructs, is more explicit in Items 21 and 22 (e.g., “Non-offending partners of individuals who have sexually offended are immoral”) when compared to item 28 (“Non-offending partners do not care about the victim of their partner’s sexual offending”). Table 2 shows standardized regression weights, standard errors, significance values and confidence intervals for the model.^2^

**Table 2:** Study 2 confirmatory factor analysis standardized regression weights for the four-factor solution (*N* = 308)

Item	Factor 1			Factor 2			Factor 3			Factor 4		
	Standardized (SE)	*p*	95% CI	Standardized (SE)	*p*	95% CI	Standardized (SE)	*p*	95% CI	Standardized (SE)	*p*	95% CI
Item 1	**.76 (.03)**	**.003**	**.69–.82**									
Item 2	**.70 (.04)**	**.003**	**.62–.77**									
Item 3	**.80 (.03)**	**.003**	**.73–.85**									
Item 4	**.68 (.04)**	**.003**	**.58–.76**									
Item 5	**.76 (.04)**	**.002**	**.67–.82**									
Item 6	**.76 (.03)**	**.002**	**.70–.82**									
Item 7	**.73 (.03)**	**.003**	**.66–.78**									
Item 8	**.64 (.05)**	**.004**	**.52–.71**									
Item 9	**.79 (.03)**	**.003**	**.73–.83**									
Item 10	**.63 (.04)**	**.002**	**.55–.71**									
Item 11	**.72 (.03)**	**.002**	**.65–.78**									
Item 12	**.64 (.04)**	**.001**	**.56–.72**									
Item 13	**.49 (.05)**	**.002**	**.38–.58**									
Item 14	**.62 (.04)**	**.002**	**.53–.69**									
Item 15				**.65 (.05)**	**.002**	**.53–.75**						
Item 16				**.63 (.05)**	**.002**	**.51–.72**						
Item 17				**.63 (.05)**	**.002**	**.52–.73**						
Item 18				**.69 (.04)**	**.001**	**.61–.77**						
Item 19				**.71 (.04)**	**.002**	**.63–.79**						
Item 20				**.79 (.03)**	**.004**	**.71–.85**						
Item 21				**.51 (.15)**	**.002**	**.20–.76**	**.35 (.14)**	**.01**	**.11–.67**			
Item 22				**.44 (.15)**	**.021**	**.07–.69**	**.43 (.14)**	**.003**	**.18–.79**			
Item 23							**.72 (.04)**	**.002**	**.64–.78**			
Item 24							**.70 (.04)**	**.002**	**.61–.77**			
Item 25							**.54 (.05)**	**.002**	**.44–.63**			
Item 26							**.79 (.04)**	**.002**	**.70–.84**			
Item 27							**.68 (.05)**	**.002**	**.57–.77**			
Item 28				.001 (.18)	.98	−.42 to .28	**.80 (.18)**	**.002**	**.52–1.26**			
Item 29							**.84 (.02)**	**.003**	**.79–.88**			
Item 30										**.95 (.02)**	**.003**	**.91–.97**
Item 31										**.92 (.14)**	**.002**	**.88–.95**

Estimator = maximum likelihood with bootstrapping (1000 samples, 95% bias-corrected confidence level). Significant (*p* < .05) regression weights are in bold. RMSEA = .053, 90% CI = .047–.060, *p* = .17; CFI = .955; TLI = .942. Standardized = standardized regression weigh

As observed during exploratory factor analysis, all four factors were again strongly significantly positively correlated. Table 3 shows correlations and covariances between all factors, as well as standard errors, significance values, and confidence intervals.

**Table 3:** Study 2 correlations and covariances between factors during confirmatory factor analysis

	Correlations			Covariances		
	Coefficient (SE)	*p*	95% CI	Coefficient (SE)	*p*	95% CI
Factor 1 <—> Factor 2	.79 (.04)	.001	.71**–**.86	0.99 (0.12)	.001	0.77**–**.1.25
Factor 1 <—> Factor 3	.68 (.04)	.002	.59**–**.76	0.91 (0.11)	.001	0.72–1.16
Factor 1 <—> Factor 4	.65 (.04)	.001	.56**–**.72	1.23 (0.13)	.001	0.97–1.49
Factor 2 <—> Factor 3	.83 (.04)	.002	.76**–**.91	0.94 (0.12)	.001	0.74–1.22
Factor 2 <—> Factor 4	.80 (.04)	.002	.70**–**.88	1.28 (0.16)	.001	0.97–1.60
Factor 3 <—> Factor 4	.81 (.04)	.002	.73**–**.88	1.38 (0.16)	.001	1.07–1.71

Estimator = maximum likelihood with bootstrapping (1000 samples, 95% bias-corrected confidence level). Factor 1 = Judgement of Relationship Decision, Factor 2 = Behavioral Intent, Factor 3 = Judgement of Character, Factor 4 = Shame

## Study 3: Scale Reliability and Discriminative Ability

The purpose of this study was to extend the findings of Study 2 through examining (1) the reliability of the APPSO when applied to a specialist sample of professionals and (2) the ability of the APPSO to discriminate between a sample of professionals working in criminal justice system-adjacent agencies and the general population. Here, we expected that those working within criminal justice system-adjacent agencies may hold more negative attitudes toward non-offending partners when compared to the general population, given that non-offending partners in previous research emphasized their negative interactions with such professionals (e.g., Armitage et al., 2024; Duncan et al., 2022; Kamitz & Gannon, 2024a).

### Method

#### Participants

We recruited 48 participants, two social workers (4.2%) and 46 police officers (95.8%), all of whom were white (*n* = 48 British).^3^ Participants’ ages ranged between 26 and 64 years (*M* = 44.60, SD = 8.36). The two social workers had been working for social services for three and seven years, respectively (*M* = 5.00, SD = 2.83), while police officers had been working for the police between one and 44 years (*M* = 19.44, SD = 9.60). Invitations for study participation and the link to the survey were sent out via email. Social workers participating in the study received a £5 voucher. Due to internal policies, police officers taking part in the study could not be financially compensated.

#### Measures

The 31-item measure was administered using Qualtrics. The internal consistency of the overall scale and each subscale/factor was “excellent” for both the general population (*α* = .96, *α*_Factor 1_ = .93, *α*_Factor 2_ = .91, *α*_Factor 3_ = .90, *α*_Factor 4_ = .93) and the professional sample (*α* = .95, *α*_Factor 1_ = .93, *α*_Factor 2_ = .82, *α*_Factor 3_ = .90, *α*_Factor 4_ = .80). Overall, those working within the police and social services had slightly positive (*M* = 2.73) attitudes toward non-offending partners, while those recruited from the general population had, overall, neither positive nor negative attitudes (*M* = 3.98). For means, medians, and standard deviations of the scale, subscales, and each item by group, see Table 4. None of the participants recruited from the police or social services failed any of the three attention checks. Eleven participants from the general population sample had already been excluded due to failed attention checks, as described during Study 2.

**Table 4:** Study 3 comparison of attitudes toward non-offending partners between professionals and the general population

	Professionals		General population		*U*	*z*	*p*	SE	*r*
	*Mdn*	*M* (SD)	*Mdn*	*M* (SD)					
**Overall scale**	**2.73**	**2.78 (0.81)**	**4.03**	**3.98 (1.07)**	**12,061.50**	**7.04**	**< .001**	**663.15**	**.37**
**Factor 1**	**3.57**	**3.45 (1.02)**	**4.69**	**4.67 (1.18)**	**11,583.50**	**6.32**	**< .001**	**663.09**	**.33**
**Item 1**	**5.00**	**4.40 (1.76)**	**6.00**	**5.35 (1.61)**	**9719.00**	**3.59**	**< .001**	**647.78**	**.19**
**Item 2**	**4.00**	**4.06 (1.77)**	**6.00**	**5.49 (1.57)**	**10,814.00**	**5.30**	**< .001**	**645.53**	**.28**
**Item 3**	**5.00**	**4.19 (1.50)**	**6.00**	**5.37 (1.46)**	**10,514.50**	**4.82**	**< .001**	**647.88**	**.26**
**Item 4**	**5.00**	**4.54 (1.57)**	**6.00**	**5.69 (1.38)**	**10,513.00**	**4.86**	**< .001**	**642.29**	**.26**
**Item 5**	**3.00**	**2.92 (1.50)**	**5.00**	**4.78 (1.82)**	**11,478.00**	**6.24**	**< .001**	**654.41**	**.33**
**Item 6**	**4.00**	**3.88 (1.36)**	**5.00**	**5.24 (1.50)**	**11,110.00**	**5.72**	**< .001**	**649.74**	**.30**
**Item 7**	**3.00**	**3.02 (1.39)**	**5.00**	**4.65 (1.70)**	**11,277.00**	**5.94**	**< .001**	**653.82**	**.31**
**Item 8**	**2.00**	**2.63 (1.20)**	**4.00**	**3.97 (1.59)**	**11,021.00**	**5.59**	**< .001**	**648.72**	**.30**
**Item 9**	**3.00**	**3.25 (1.38)**	**5.00**	**4.60 (1.72)**	**10,671.50**	**5.01**	**< .001**	**654.48**	**.27**
**Item 10**	**4.00**	**4.06 (1.49)**	**6.00**	**5.49 (1.38)**	**11,222.00**	**5.92**	**< .001**	**646.84**	**.31**
**Item 11**	**2.00**	**2.69 (1.29)**	**4.00**	**3.75 (1.76)**	**10,049.00**	**4.07**	**< .001**	**652.874**	**.22**
**Item 12**	**2.00**	**2.54 (1.11)**	**3.00**	**3.56 (1.58)**	**10,601.50**	**4.99**	**< .001**	**643.08**	**.26**
**Item 13**	**2.00**	**2.23 (1.33)**	**4.00**	**3.76 (1.81)**	**11,075.50**	**5.63**	**< .001**	**654.45**	**.30**
**Item 14**	**4.00**	**3.92 (1.35)**	**5.00**	**4.48 (1.39)**	**9145.00**	**2.72**	**.007**	**644.56**	**.14**
**Factor 2**	**2.44**	**2.45 (0.83)**	**3.50**	**3.47 (1.21)**	**11,214.00**	**5.77**	**< .001**	**662.76**	**.31**
**Item 15**	**2.00**	**2.50 (1.38)**	**3.00**	**3.63 (1.57)**	**10,661.00**	**5.03**	**< .001**	**650.41**	**.27**
**Item 16**	**2.00**	**2.50 (1.01)**	**3.00**	**3.59 (1.58)**	**10,487.00**	**4.78**	**< .001**	**647.99**	**.25**
**Item 17**	**2.00**	**2.27 (1.23)**	**3.00**	**2.83 (1.37)**	**9181.00**	**2.77**	**.006**	**644.87**	**.15**
Item 18	4.00	3.94 (1.78)	5.00	4.40 (1.75)	8457.00	1.63	.104	654.33	.09
**Item 19**	**3.00**	**3.21 (1.54)**	**5.00**	**4.60 (1.64)**	**10,776.00**	**5.19**	**< .001**	**652.36**	**.28**
**Item 20**	**2.00**	**1.75 (0.98)**	**3.00**	**2.85 (1.53)**	**10,699.50**	**5.12**	**< .001**	**646.20**	**.27**
**Item 21**	**2.00**	**1.90 (1.10)**	**3.00**	**3.18 (1.60)**	**11,008.00**	**5.58**	**< .001**	**647.97**	**.30**
**Item 22**	**1.00**	**1.56 (0.68)**		**2.70 (1.44)**	**11,038.50**	**5.68**	**< .001**	**642.13**	**.30**
**Factor 3**	**2.00**	**1.98 (0.77)**	**3.14**	**3.17 (1.22)**	**11,624.00**	**6.39**	**< .001**	**662.65**	**.34**
**Item 23**	**2.00**	**1.79 (0.99)**	**2.00**	**2.71 (1.53)**	**10,012.50**	**4.08**	**< .001**	**642.58**	**.22**
**Item 24**	**1.00**	**1.60 (0.82)**	**2.00**	**2.69 (1.43)**	**10,766.00**	**5.25**	**< .001**	**643.16**	**.28**
**Item 25**	**2.00**	**2.50 (1.15)**	**4.00**	**3.74 (1.50)**	**10,855.50**	**5.35**	**< .001**	**647.71**	**.28**
**Item 26**	**2.00**	**1.83 (0.81)**	**4.00**	**3.62 (1.68)**	**11,905.00**	**6.91**	**< .001**	**653.33**	**.37**
**Item 27**	**2.00**	**2.10 (1.13)**	**2.00**	**2.85 (1.40)**	**9671.00**	**3.55**	**< .001**	**642.20**	**.19**
**Item 28**	**2.00**	**2.08 (1.01)**	**3.00**	**3.31 (1.63)**	**10,642.00**	**5.00**	**< .001**	**649.96**	**.26**
**Item 29**	**2.00**	**1.96 (0.90)**	**3.00**	**3.24 (1.65)**	**10,761.50**	**5.19**	**< .001**	**649.75**	**.28**
**Factor 4**	**2.00**	**2.14 (1.15)**	**4.00**	**3.58 (1.64)**	**11,158.00**	**5.72**	**< .001**	**658.32**	**.30**
**Item 30**	**2.00**	**2.10 (1.29)**	**4.00**	**3.42 (1.65)**	**10,811.50**	**5.25**	**< .001**	**651.54**	**.28**
**Item 31**	**2.00**	**2.17 (1.23)**	**4.00**	**3.75 (1.74)**	**11,188.50**	**5.81**	**< .001**	**653.65**	**.31**

Professionals = participants working for the police or social services (*n* = 48), General Population = participants recruited from Prolific (*n* = 308). Factor 1 = Judgement of Relationship Decision, Factor 2 = Behavioral Intent, Factor 3 = Judgement of Character, Factor 4 = Shame. Higher scores = more negative attitudes toward non-offending partners. All significant differences between the two groups are in bold. Items 12, 15, 16, and 17 were reverse-coded

### Results

IBM SPSS Statistics 28 (IBM Corp., 2021) was used for all analyses. As established previously, Pearson’s zero-order correlation coefficient *r* was interpreted according to Funder and Ozer’s (2019) guidelines.

Shapiro–Wilk tests indicated that while the data were normally distributed for the overall scale, they were not normally distributed for the factors and individual items. Because of this, we conducted Mann–Whitney U tests to assess whether attitudes toward non-offending partners, as evaluated by the items, subscales/factors and overall measure differed significantly between the general population and professionals working within the police and social services. Overall, those working within the police and social services had more positive attitudes toward non-offending partners than participants in the general population (*U* = 12,061.50, *p* < .001). This effect was large (*r* = .37). Additionally, police officers and social workers were less judgemental of non-offending partners’ decision to stay with their partner who has offended (Factor 1; *U* = 11,583.50, *p* < .001), had more positive behavioral intentions toward non-offending partners (Factor 2; *U* = 11,214.00, *p* < .001), judged non-offending partners’ characters more positively (Factor 3; *U* = 11,624.00, *p* < .001), and shamed non-offending partners less (Factor 4; *U* = 11,158.00, *p* < .001) than participants from the general population. These effects were also large (*r*_F1_ = .33, *r*_F2_ = .31, *r*_F3_ = .34, *r*_F4_ = .31). A similar trend emerged for most of the individual items: we found differences in item scores between professionals and the general public, ranging from small (Item 14: “Non-offending partners are in denial about their partners’ sexual offending”, *r* = .14) to large (Item 26: “Non-offending partners of people who have sexually offended are weak(-minded)”, *r* = .37). Here, those working within the police or social services had, on average, moderately more positive attitudes toward non-offending partners (i.e., scored lower on the items) than those recruited from the general population (*r* = .26). The only exception to this was Item 18 (“I would distance myself from a friend if I found out that they were in a relationship with someone who has sexually offended”). While police officers and social workers indicated lower agreement with this item than participants from the general population, this difference was not significant (*U* = 8457.00, *p* = .104). See Table 4 for means, medians and standard deviations for both groups, as well as all relevant test statistics.

## Study 4: Online Survey-Based Validation

The aim of this study was to validate the APPSO, using an online general population sample. This was achieved through a two-pronged approach: (1) evaluating the APPSO’s construct validity by assessing its associations with theoretically relevant variables (i.e., demographic characteristics, political views, characteristics relating to sexual violence) and (2) assessing the APPSO’s criterion-related validity by investigating its ability to predict participants’ intent to discriminate against or support non-offending partners.

The variables chosen to assess content validity spanned demographic characteristics, ideological beliefs, personal experiences, and related attitudes. Demographic factors (i.e., education, income, presence of children in the household) have been shown to influence attitudes relevant to sexual offending (Willis et al., 2013). Additionally, ideological beliefs (i.e., conservatism, feminism, and party affiliation) have been demonstrated to shape beliefs about justice, personal responsibility, and gendered violence (Flood & Pease, 2009; Payne et al., 2004). Personal experience variables (i.e., history of sexual victimization/being a non-offending partner) may influence attitudes toward non-offending partners, with research on intergroup contact and stigma suggesting that individuals with personal or vicarious experience of stigma may express more empathetic attitudes toward similarly affected groups (Pettigrew & Tropp, 2006).

### Method

#### Participants

We included a sample of 350 Prolific users (female: 48.6%, male: 49.7%, other: 1.7%) in the final analysis. All participants resided in the UK, and participants’ ages ranged between 18 and 77 years (*M* = 39.33, SD = 12.20). Most participants were white (*n* = 290, 82.9%), while 6.3% (*n* = 22) were Asian or Asian British, 6.3% (*n* = 22) were Black, Black British, Caribbean or African, and 3.1% (*n* = 11) were from two or more ethnic groups. Five participants (1.4%) indicated that they belonged to an ethnic group not listed.

#### Design

To assess the APPSO’s construct validity, this research followed a correlational design to explore the relationships between participants’ scores on the entire APPSO scale and each of its subscales, and participants’ demographic characteristics (age, gender, ethnicity, education level, household income, children in the household), political views and affiliation (conservatism, feminism, party affiliation), sexual offense victimization, non-offending partner status, and attitudes toward those who have sexually offended. To examine the APPSO’s criterion-related validity, a regression-based design assessed which of the characteristics mentioned above (including APPSO total and subscales) predict individual-level behaviors, such as intent to discriminate, and support for policy affecting non-offending partners.

#### Measures

*Education Level*. Participants were asked to indicate their highest level of qualification with answer options taken from the Census 2021 for England (Office for National Statistics, n.d.) and ranked from lowest to highest level of education (i.e., [0] No formal qualifications, [1] One to four GCSEs (grade A* to C or 4+) or equivalent, [2] Five or more GCSEs (grade A* to C or 4+) or equivalent, [3] Apprenticeships, [4] Two or more A Levels or equivalent, [5] Higher National Certificate/Diploma, Bachelor’s degree or postgraduate qualification, [NA] Other qualifications of unknown level). Those indicating that they had other qualifications of an unknown level where excluded from analyses involving this variable.

*Income*. Participants were asked to report their household’s average weekly income.

In line with the 2022–2023 Family Resources Survey (Department for Work and Pensions, 2024), answer options ranged between “£200” and “£2000 or more”, with rising increments of £199 (i.e., “*£200–£399*”).

*Children in Household*. To assess whether participants currently live in the same household as any children, participants were asked to respond to “Are any children under the age of 18 currently part of your household?” with either “Yes” or “No”.

*Conservatism*. To measure participants’ level of political conservatism, participants were asked to respond to the single item “Politically, I identify as conservative” on a 7-point Likert-type scale (1 = *Strongly disagree*, 7 = *Strongly agree*). Thus, higher scores indicated higher levels of political conservatism.

*Feminism*. To measure participants’ self-identification as a feminist, participants were asked to respond to the single item “I am a feminist” on a 7-point Likert-type scale (1 = *Strongly disagree*, 7 = *Strongly agree*). Thus, higher scores indicated higher levels of feminist self-identification.

*Party Affiliation*. Participants’ political party affiliation was measured by asking participants “Which party did you vote for during the last general election?” Here, participants could respond with either of the four parties which received the most votes during the 2024 UK general election or indicate that they voted for a different party or did not vote. The parties listed were the Labour party (i.e., center-left social democrats; current governing party), the Conservative party (i.e., center-right to right-wing conservatists), Reform UK (i.e., right-wing populists), and the Liberal Democrats (i.e., center to center-left liberalists). Parties that can only be voted for in parts of the UK (e.g., Sinn Féin, the Scottish National Party) were not explicitly listed despite attracting more votes than some listed parties.

*Victim Status*. To measure if participants had previously been a victim of sexual offending, participants were asked to respond to the single question “Are you a victim/survivor of sexual offending?” with “Yes” or “No”. Given the sensitive nature of the question, participants could also choose not to disclose their victim status.

*Non-Offending Partner Status*. To measure if participants are currently, or have ever been, a non-offending partner of someone who has sexually offended, participants were asked to respond to the single question “Has a current or ex-partner of yours ever committed, or been accused of committing, a sexual offense?”, with “Yes” or “No”. Given the sensitive nature of the question, participants could also choose not to answer this question.

*Attitudes Toward Those who Have Sexually Offended*. Participants’ attitudes toward those who have sexually offended was assessed using the Community Attitudes Toward Sex Offenders (CATSO) Scale (Church et al., 2008). Participants responded to a total of 18 items (e.g., “Convicted sex offenders should never be released from prison”) on a 6-point Likert-type scale (1 = *Strongly disagree*, 6 = *Strongly agree*). Averaged scores range from 1 to 6, with higher scores indicating more negative and stereotypical attitudes toward those who have sexually offended. In our study, the overall scale demonstrated “moderate” internal consistency (*α* = .76).

*Attitudes Toward Non-Offending Partners*. As previously, the 31-item APPSO was administered to assess participants’ attitudes toward non-offending partners of those who have sexually offended. Once again, participants responded to the items on a 7-point Likert-type scale (1 = Fully disagree, 7 = Fully agree). Averaged scores ranged from 1 to 7, with higher scores indicating more negative attitudes toward non-offending partners. The overall scale (*α* = .96), and each of the subscales—Judgement of Relationship Decision (*α* = .94), Behavioral Intent (*α* = .91), Judgement of Character (*α* = .90), and Shame (*α* = .90)—demonstrated “excellent” internal consistency.

*Intent to Discriminate Against Non-Offending Partners*. Participants’ intent to discriminate against or, on the other hand, support, non-offending partners of those who have committed a sexual offense was assessed using a number of different vignettes. These placed participants in scenarios in which they might encounter non-offending partners in real life, corresponding to areas of hardship non-offending partners commonly face: In situations related to housing, workplace or financial situations, and lastly social and support situations (see Table 5 for the full vignettes). Participants responded to each of the scenarios using a 7-point Likert-type scale. Here, lower scores indicated intent to behave more negatively and discriminatory toward non-offending partners.

**Table 5:** Study 4 vignettes measuring intent to discriminate against non-offending partners

Housing	Imagine that you are a landlord who owns property. How likely would you be renting a flat to a non-offending partner if they applied to live in the flat by themselves?
	How comfortable would you be living next door to a non-offending partner who is living alone?
	How likely would you be to support a government policy that aims to provide stable housing to non-offending partners?
Financial/workplace	Imagine that you are in a position to make hiring decisions. How likely would you be to hire a non-offending partner… … for a job that involves childcare (e.g., nursery teacher or babysitter)? … for a job in healthcare (e.g., nurse or doctor)? …for a job in a factory?
	If you found out that one of your work colleagues’ partners committed a sexual offense, how comfortable would you feel working alongside this colleague in the future?
	How likely would you be to support a government policy that provides financial support for non-offending partners in need?
Social support and ostracism	How likely would you be to continue a friendship after finding out that your friend’s partner has committed a sexual offense?
	How likely would you be to continue a relationship with a family member after finding out that their partner has committed a sexual offense?
	If you found out that a family member or friend was a non-offending partner, how comfortable would you feel with them looking after your children, by themselves without the person who has offended present? If you do not have any children, please imagine that you do
	How likely would you be to support a government policy that would provide the police and social services with resources to help non-offending partners?

#### Procedure

The study was advertised on Prolific on 30 of July 2024 as “Measuring Attitudes Towards Non-Offending Partners of Individuals who Have Sexually Offended.” Participants who had taken part in any of the previous studies as part of this research were automatically excluded from participating. The questionnaire was administered using Qualtrics. Here, participants first reported their demographic characteristics (age, gender, ethnicity, education level, household income, children in household) before answering questions regarding their political views (conservatism and feminism) and their political party affiliation. Participants subsequently reported any sexual offense victimization as well as their current or previous non-offending partner status. Then, participants completed the CATSO as a measure of their attitudes toward those who have sexually offended, before completing the 31-item APPSO. Finally, participants were presented with and responded to the vignettes. Once again, participants failing two or more of the three attention checks (*n* = 7) were excluded from analysis, in line with Prolific’s attention check policy (Prolific, 2022). Participants received £1.05 for their participation.

### Results

IBM SPSS Statistics 28 (IBM Corp., 2021) was used for all analyses. As established previously, internal consistency coefficient *α* was interpreted according to Ponterotto and Ruckdeschel’s (2007) guidelines, and correlation coefficients were interpreted according to Funder and Ozer’s (2019) guidelines.

*Relationships Between Attitudes Toward Non-Offending Partners and Other Factors*. Relationships between APPSO scores and variables including age, education, income, conservatism, feminism, and attitudes toward those who have sexually offended were assessed using correlation.^4^^,^^5^ Independent samples *t*-tests examined differences in APPSO scores by gender, cohabitating with a child, victim/survivor status, and experience as a non-offending partner.^6^ One-way ANOVAs assessed differences by ethnic group and political affiliation.^7^ Due to low *n,* participants from multiple ethnic groups (*n* = 11) and those who indicated belonging to an ethnic group not listed (*n* = 5) were excluded from the analysis. Given the lack of political consistency, participants who indicated that they voted for a political party not listed were also excluded. For correlational analysis statistics for these variables, see Table 6.

**Table 6:** Study 4 relationships between attitudes toward non-offending partners and other variables

	APPSO total		Judgement of relationship decision		Behavioral intent		Judgement of character		Shame	
	*r*/ρ	95% CI	*r*/ρ	95% CI	*r*/ρ	95% CI	*r*/ρ	95% CI	*r*/ρ	95% CI
Age	−.17**	**−.27; −.06**	−.11*	**−.21; −.01**	−.21***	**−.31; −.11**	−.16**	**−.26; −.05**	−.12*	**−.22; −.02**
Level of education	−.20***	**−.30; −.09**	−.17***	**−.27; −.07**	−.18***	**−.28; −.07**	−.18***	**−.28; −.07**	−.13*	**−.23; −.02**
Household income	**−**.08	**−**.18; .02	**−**.06	**−**.17; .04	**−**.10	**−**.21; .00	**−**.07	**−**.18; .03	**−**.03	**−**.13; .08
Conservatism	.06	**−**.05; .16	.05	**−**.06; .15	.04	**−**.06; .15	.06	**−**.04; .17	.08	**−**.03; 1.8
Feminism	−.14**	**−.24; −.04**	−.11*	**−.21; −.01**	−.14**	**−.25; −.04**	−.12*	**−.23; −.02**	−.17*	**−.22; −.01**
Attitudes toward those who have sexually offended	.57***	**.50; .64**	.51***	**.43; .58**	.46***	**.38; .54**	.56***	**.49; .63**	.46***	**.38; .54**

For continuous variables, (i.e., APPSO total and all subscales, age, conservatism, feminism, and attitudes toward those who have sexually offended), Pearson correlation coefficient *r* is reported. For ordinal variables (i.e., level of education and household income), Spearman’s rank correlation coefficient *ρ* is reported

***p ≤ .001; **p ≤ .01; *≤ .05. Significant correlations are in bold

*Demographic Characteristics.* Older participants and those with higher levels of education reported slightly-to-moderately and moderately more positive attitudes toward non-offending partners, respectively. Additionally, women (*M* = 4.00, SD = 1.04) held slightly more negative attitudes toward non-offending partners than men [*M* = 3.67, SD = 1.0; *t*(342) = − 2.89, *p* = .004, 95% CI (− .54, − .10)]. Ethnic group differences were small, with white participants expressing more positive attitudes than Asian or Asian British participants [*F*(2, 331) = 3.00, *p* = .051, *η*^2^ = .02]. However, no significant relationships were found between overall attitudes toward non-offending partners and weekly household income, as well as whether participants lived in the same household as a child. However, those with a child in the household (*M* = 3.83, SD = 1.69) reported shaming non-offending partners slightly more than those who did not live with a child [*M* = 3.43, SD = 1.57; *t*(348) = 2.23, *p* = .014, 95% CI (.05, .74), *d* = .24].

*Political Orientation.* There was no significant relationship between conservatism and attitudes toward non-offending partners. However, higher levels of feminism were slightly-to-moderately associated with a more positive attitudes toward non-offending partners. We also found medium-sized significant differences in attitudes toward non-offending partners depending on political party affiliation [*F*(4, 306) = 4.55, *p* < .001, *η*^2^ = .06]. Specifically, those who voted for the Liberal Democrats had more positive attitudes toward non-offending partners compared to voters of other parties.

*Factors Related to Sexual Offending.* No significant APPSO score differences were found by victim/survivor status or personal experience as a non-offending partner. However, participants with more negative attitudes toward those who have sexually offended similarly reported more negative attitudes toward non-offending partners, a very large effect.

*Intent to Discriminate*. Regression analyses assessed whether APPSO scores predicted individual-level behaviors toward non-offending partners, such as intent to discriminate (regarding finances/work and housing), and support for policy affecting non-offending partners. Regression statistics for analyses predicting intent to discriminate from total APPSO scores in each vignette condition, see Table 7.^8^

**Table 7:** Study 4 regression results predicting intent to discriminate from total APPSO scores

*Housing discrimination*					
Predictor	*B*	*β*	SE	*t*	*p*
	Outcome: likelihood to rent (*M* = 5.43, SD = 1.51)				
Intercept	7.80		0.27	29.03	< .001
Model 1: Total APPSO scale	**−**0.64	**−**0.45	0.07	**−**9.31	< .001
	Outcome: comfort living next door (*M* = 5.21, SD = 1.60)				
Intercept	8.01		0.28	29.59	< .001
Model 1: Total APPSO scale	**−**0.74	**−**0.49	0.07	**−**10.38	< .001
	Outcome: support for housing policy (*M* = 5.37, SD = 1.47)				
Intercept	8.13		0.25	32.31	< .001
Model 1: Total APPSO scale	**−**0.72	**−**0.52	0.06	**−**11.37	< .001
*Financial/workplace discrimination*					
	Outcome: hiring for childcare (*M* = 4.02, SD = 1.88)				
Intercept	7.8		0.31	24.94	< .001
Model 1: Total APPSO scale	**−**0.99	**−**0.56	0.08	**−**12.53	< .001
	Outcome: hiring for healthcare (*M* = 4.41, SD = 1.81)				
Intercept	8.26		0.29	28.32	< .001
Model 1: Total APPSO scale	**−**1.01	**−**0.59	0.07	**−**13.69	< .001
	Outcome: hiring for factory work (*M* = 5.61*, *SD = 1.19)				
Intercept	7.36		0.22	33.84	< .001
Model 1: Total APPSO scale	**−**0.46	**−**0.41	0.06	**−**8.31	< .001
	Outcome: comfort working alongside (*M* = 3.91, SD = 1.76)				
Intercept	7.42		0.29	25.25	< .001
Model 1: Total APPSO scale	**−**0.92	**−**0.55	0.07	**−**12.39	< .001
	Outcome: support for financial policy (*M* = 4.73, SD = 1.56)				
Intercept	7.91		0.26	30.6	< .001
Model 1: Total APPSO scale	**−**0.83	**−**0.56	0.07	**−**12.74	< .001
*Social support/ostracism*					
	Outcome: likelihood of continuing friendship (*M* = 4.39, SD = 1.70)				
Intercept	8.41		0.26	32.87	< .001
Model 1: Total APPSO scale	**−**1.06	**−**0.66	0.07	**−**16.32	< .001
	Outcome: likelihood of continuing family relationship (*M* = 4.55, SD = 1.75)				
Intercept	8.69		0.27	32.78	< .001
Model 1: Total APPSO scale	**−**1.09	**−**0.66	0.07	**−**16.21	< .001
	Outcome: comfort of non-offending partner looking after child (*M* = 3.82, SD = 1.84)				
Intercept	7.79		0.30	26.39	< .001
Model 1: Total APPSO scale	**−**1.04	**−**0.60	0.08	**−**13.97	< .001
	Outcome: support for policy providing support (*M* = 5.10, SD = 1.45)				
Intercept	7.83		0.25	31.76	< .001
Model 1: Total APPSO scale	**−**0.72	**−**0.53	0.06	**−**11.51	< .001

*Housing.* Total APPSO scores significantly predicted willingness to rent to [*F*(1, 348) = 86.67, *p* < .001, *R*^2^ = 19.9%], live next-door to a non-offending partner [*F*(1, 348) = 107.79, *p* < .001, *R*^2^ = 23.6%], or support policy aimed at providing housing to non-offending partners [*F*(1, 348) = 129.18, *p* < .001, *R*^2^ = 27.1%]. Including APPSO subscales as predictors in the model improved model fit (*R*^2^ = 27.3%/31.1%/36.8%), with Behavioral Intent consistently the strongest predictor.

*Financial/Workplace.* Total APPSO scores significantly predicted participants’ likelihood of hiring a non-offending partner for a job in childcare [*F*(1, 348) = 157.10, *p* < .001, *R*^2^ = 31.1%], healthcare [*F*(1, 348) = 187.15, *p* < .001, *R*^2^ = 35.0%], and in a factory [*F*(1, 348) = 69.06, *p* < .001, *R*^2^ = 16.6%]. Including APPSO subscales as predictors in the model again improved the model fit (*R*^2^ = 33.9%/40.0%/21.5%), with Behavioral Intent the strongest predictor within each of these models. Additionally, APPSO scores significantly predicted participants’ levels of comfort working alongside a non-offending partner [*F*(1, 348) = 153.43, *p* < .001, *R*^2^ = 30.6%], as well as their support for a government policy providing financial support for non-offending partners in need [*F*(1, 348) = 162.31, *p* < .001, *R*^2^ = 31.8%]. Including APPSO subscales as predictors improved the model fit (*R*^2^ = 33.5%/38.7%), with Behavioral Intent as the strongest predictor in both models.

*Social Support and Ostracism.* Total APPSO scores significantly predicted participants’ likelihood of continuing a friendship [*F*(1, 348) = 266.20, *p* < .001, *R*^2^ = 43.3%] or a family relationship with a non-offending partner [*F*(1, 348) = 262.84, *p* < .001, *R*^2^ = 43.0%], as well as participants’ comfort with having a non-offending partner look after their, the participant’s, child [*F*(1, 348) = 195.21, *p* < .001, *R*^2^ = 35.9%], and support for a government policy providing support resources for non-offending partners through the police and social services [*F*(1, 348) = 132.38, *p* < .001, *R*^2^ = 27.6%]. As with the previous vignette scenarios, including APPSO subscales as predictors in the model improved the model fit (*R*^2^ = 53.0%/53.7%/38.6%/36.6%), with Behavioral Intent consistently the strongest predictor.

## Study 5: Laboratory-Based Validation

This final study aimed to enhance the ecological validity of the APPSO’s validation through a laboratory-based approach. Using the hot sauce paradigm (Lieberman et al., 1999) to measure behavioral aggression, we investigated whether more negative attitudes toward non-offending partners predicted increased aggression toward this group. We hypothesized that more negative attitudes toward non-offending partners, as measured by higher scores on the APPSO, would predict higher levels of aggression toward non-offending partners, but not other individuals.

### Method

#### Participants

Participants were recruited through advertisements on news boards, local social media groups, and email newsletters in a UK university city. We included a final sample of 58 participants (female: 70.7%, male: 24.1%, other: 5.2%) in the final analysis. Participants’ ages ranged between 18 and 58 years (*M* = 22.89, SD = 6.73; one participant did not provide their age). Most participants were white (*n* = 24, 41.4%), while 31.0% (*n* = 18) were Asian or Asian British, 19.0% (*n* = 11) were Black, Black British, Caribbean, or African, and 5.2% (*n* = 3). Two participants (3.4%) indicated that they belonged to an ethnic group not listed. Chi-square tests and an independent samples *t*-test showed that there was no significant difference in demographic factors between the experimental and control conditions [gender: *χ*^2^(2) = 4.69, *p* = .096; ethnicity: *χ*^2^(4) = 1.91, *p* = .752; age: *t*(55) = .624, *p* = .535].

#### Design

This study employed a between-participant experimental design to assess whether negative attitudes toward non-offending partners, as measured by higher scores on the APPSO, would predict higher levels of behavioral aggression toward non-offending partners but not toward other individuals. Participants were randomly assigned to either the experimental condition, in which they were paired with a fictitious “other participant” who disclosed their status as a non-offending partner, or the control condition, where the fictitious participant revealed that their partner had been unfaithful. Following the hot sauce paradigm (Lieberman et al., 1999), the dependent variable was the quantity of hot sauce participants allocated to the fictitious “other participant,” which served as a measure of aggression. We hypothesized that higher APPSO scores would predict greater aggression (i.e., larger amount of hot sauce allocated) in the experimental condition but not in the control condition.

#### Measures

This experimental study followed Lieberman et al. (1999) hot sauce allocation paradigm. Upon arrival, participants were informed that they would complete two separate studies: One focusing on personality and writing styles and another study focusing on taste preferences. First, participants completed a short demographic questionnaire and the APPSO scale.^9^ Afterward, they filled in the Big Five Personality Inventory (John et al., 2008) to distract them from the link between the APPSO’s contents and the “other participant’s” identity as a non-offending partner. Participants were then asked to write a short paragraph about a recent impactful even in their lives and, following this, received a handwritten paragraph ostensibly written by another, unbeknownst to them, fictitious, participant (hereafter referred to as Participant B). In the experimental condition, Participant B’s paragraph revealed their status as a non-offending partner of someone who had accessed child sexual abuse materials online, as follows:

I recently had an experience that really impacted me. I’ve been dating my boyfriend for about a year now, and we moved in together in May. Everything in our relationship was going really well. But then about 2 weeks ago, *the police knocked on our door out of the blue. I had no idea what they were there for, but they wouldn’t tell me and just said that I should ask my boyfriend. I thought this must be a misunderstanding. But when I confronted him, he confessed and said that he looked at some indecent images of teenagers online because he found them on a porn site.* He moved out of our flat but I’m not sure how to deal with the situation because he said that it only happened once, and he won’t do it again.

In the control condition, the vignette was changed so that Participant B disclosed that their partner had been unfaithful, by changing the wording between the asterisks as follows:

*this woman knocked on our door out of the blue. I had no idea what she was there for, but she wouldn’t tell me and just said that I should ask my boyfriend. I thought this must be a misunderstanding. But when I confronted him, he confessed and said that he had slept with someone who he met on a dating app.*

To ensure engagement with the materials, participants were asked to rate the quality of Participant B’s writing on a 10-point scale (1 = *Extremely poor*, 10 = *Extremely good*), and were told that Participant B would also rate their written paragraph. After this, participants were informed that they had now completed the first study and were now to begin the second study focusing on taste preferences. First, participants completed a Taste Preference Inventory, rating their liking for various flavors (e.g., dry, salty, spicy) on a 21-point scale (1 = *No liking at all*; 21 = *Extreme liking*). Participants were then informed that they had been assigned to the “dry food condition” and received a bland cracker in a sealed envelope that they were told was allocated to them by Participant B as a taste sample. They were instructed to taste the cracker after the experimenter left the room and to rate their liking of it on a 9-point scale (1 = *No liking at all*, 9 = *Extreme liking*).

Participants were then informed that Participant B had been assigned to the “spicy food condition” and were shown Participant B’s (fictitious) Taste Preference Inventory, which indicated a strong dislike for spicy food. Participants were asked to allocate a quantity of medium-hot sauce for Participant B, which they were told the participant would have to consume in its entirety. Before allocating the sauce, participants were instructed to taste the hot sauce themselves and were provided with water to emphasize the spiciness of the sauce and potential unpleasantness of having to taste it. Once the experimenter had left the room, participants placed the allocated amount of hot sauce into a graduated cylinder, labeled it with Participant B’s participant number, and placed it under a cloth—ostensibly to preserve the blindness of the experimenter. To standardize the procedure, participants received a checklist to guide their actions during the hot sauce allocation.

After the experimenter collected the hot sauce sample, participants completed a manipulation check asking them how much the other participant, who they allocated food to, likes this kind of food (1 = *Not at all*, 5 = *Extremely*). Finally, participants were fully debriefed, with the deceptive aspects of the study explained thoroughly. Participants were reimbursed with a £10 online voucher.

Of the original 70 participants, three participants failed the manipulation check and indicated that Participant B enjoyed or somewhat enjoyed spicy foods. These participants were excluded as their allocation decisions were subsequently not based on the fact that Participant B did not enjoy spicy food and thus did not reflect aggression. Given that all other participants passed the manipulation checks, no participant was excluded due to overly large or small quantities of hot sauce being allocated. One participant informed the experimenter after completing the experiment that they had not understood the survey questions due to a language barrier and was subsequently also excluded from final analysis. An additional 8 participant reported doubts about the true nature of the experiment or the existence of the “other participants.” As a result, these participants were also excluded from the final analysis. Participants who were excluded from the final analysis still received the full compensation.

### Results

IBM SPSS Statistics 28 (IBM Corp., 2021) was used for all analyses. As established previously, internal consistency coefficient *α* was interpreted according to Ponterotto and Ruckdeschel’s (2007) guidelines, and correlation coefficients were interpreted according to Funder and Ozer’s (2019) guidelines.

A sensitivity analysis showed that, with 80% power and at *α* = .05, the minimum effect size detectable for a linear regression was small to medium (*f*^2^ = .14). An independent samples t-test revealed no significant difference in hot sauce allocation between the experimental (*M* = 5.00, SD = 9.24) and control (M = 3.66, SD = 2.96) conditions [*t*(56) = 0.76, *p* = .452]. Correlation analyses revealed no significant relationships between participants’ APPSO total scores and the amount of hot sauce allocated in both the experimental (*r* = .23, *p* = .186) and control conditions (*r* = .11, *p* = .554). We also did not observe a relationship between any of the APPSO subscales and the amount of hot sauce allocated in the experimental (Judgement of Relationship Decision: *r* = .15, *p* = .455; Behavioral Intent: *r* = .28, *p* = .152; Judgement of Character: *r* = .31, *p* = .113; Shame: *r* = .35, *p* = .070) or control conditions (Judgement of Relationship Decision: *r* = 13, *p* = .498; Behavioral Intent: *r* = −.02, *p* = .901; Judgement of Character: *r* = .06, *p* = .754; Shame: *r* = .29, *p* = .12). Because of an absence of a clear relationship between APPSO scores and the amount of hot sauce allocated, as well as a violation of the assumption of linearity in the data, the pre-registered linear regression analysis was not conducted. These findings indicate that negative attitudes toward non-offending partners, as measured by the APPSO and its subscales, did not clearly predict or relate to behavioral aggression in the form of hot sauce allocation under these experimental conditions. For descriptive statistics by condition regarding the APPSO total and subscale scores, and the amount of hot sauce allocated, see Table 8.

**Table 8:** Study 5 descriptive statistics by condition

Variable	Condition					
	Experimental (*n* = 28)		Control (*n* = 30)		Overall sample (*N* = 58)	
	*M* (SD)	95% CI	*M* (SD)	95% CI	*M* (SD)	95% CI
Attitudes toward non-offending partners^a^	3.55 (1.00)	3.16,3.94	4.15 (0.80)	3.85,4.44	3.86 (0.95)	3.61,4.11
Judgement of relationship decision^a^	4.11 (1.20)	3.64,4.57	4.71 (0.93)	4.36,5.06	4.42 (1.10)	4.13,4.71
Behavioral intent^a^	3.08 (1.02)	2.68,3.48	3.53 (1.05)	3.14,3.92	3.31 (1.05)	3.04,3.59
Judgement of character^a^	3.05 (1.04)	2.65,3.45	3.70 (0.98)	3.34,4.07	3.39 (1.06)	3.11,3.67
Shame^a^	3.25 (1.44)	2.69,3.81	4.18 (1.57)	3.60,4.77	3.73 (1.57)	3.32,4.15
Hot sauce allocated^b^	5.01 (9.24)	1.43,8.59	3.66 (2.96)	2.56,4.77	4.31 (6.73)	2.54,6.08

^a^Range = 1–7

^b^Range = 0 ml–50ml

Confidence internal displayed is the confidence interval for the mean

## Discussion

The aim of the current studies was to develop and validate the APPSO as a questionnaire to measure attitudes toward non-offending partners. The findings, across five studies, indicate robust and consistent support for the APPSO and its four constructs. Study 1 showed that the APPSO had robust construct validity and internal reliability of subscales via exploratory factor analysis and associated analysis of psychometric properties. Study 2 indicated, via confirmatory factor analysis, that the APPSO was a reliable, valid, and coherent measure of attitudes toward non-offending partners. Study 3 demonstrated that professionals working for the police and social services, who may encounter non-offending partners in a professional capacity, hold more favorable attitudes toward non-offending partners, when compared to a general population sample. This indicates that the APPSO and its subscales hold discriminatory validity. Study 4 demonstrated the APPSO’s construct and criterion-related validity, with findings highlighting the predictive power of attitudes toward non-offending partners in determining intent to discriminate against or support this population. Lastly, Study 5 aimed to enhance the ecological validity of the previous findings by assessing whether scores on the APPSO predict behavioral aggression toward non-offending partners. However, despite having adequate power to detect a medium effect, no significant relationship was found.

To our knowledge, the APPSO is the first measure assessing attitudes toward non-offending partners which differs from and improves upon a previous scale developed by Plogher et al. (2016) in a variety of ways: First, our scale was specifically developed to assess attitudes toward all non-offending partners of those who have committed sexual offenses, rather than being developed with a predominant focus on a small subset of non-offending partners (i.e., partners of those on the registry). This makes usage of the APPSO scale more generalizable across varying cultural and legislative contexts. Second, item development and factor analyses of our scale were informed not only by a general population sample, but by non-offending partners themselves as well as professionals likely to encounter non-offending partners as part of their work. This ensures greater content validity of the scale. Third, the scale developed in the current study used sufficiently large samples for both exploratory and confirmatory factor analysis which ensures the replicability and thus generalizability of the factor structure observed in the current study (see Boateng et al., 2018). Lastly, the APPSO scale uses non-stigmatizing, person-centered language to avoid further stigmatization and traumatization of non-offending partners which may inadvertently occur as a result of labeling.

In the following section, we interpret the structure of the APPSO scale and the study’s other findings in light of arising theoretical implications. We then outline potential practical implications as well as avenues for future research.

### Findings and Theoretical Implications

Internal consistency coefficients throughout indicated that the 31 items formed a coherent measure across multiple populations. The scale appeared to measure four main constructs: Judgement of Relationship Decision, Behavioral Intent, Judgement of Character, and Shame. The distinctiveness of these constructs was confirmed through confirmatory factor analysis with an independent population. Judgement of Non-Offending Partners’ Relationship Decision (e.g., “I don’t understand how someone could possibly choose to stay in a relationship with a person who has sexually offended”) supports the findings of previous research in which non-offending partners (Kamitz & Gannon, 2024a), and those in the general population who imagined themselves in a scenario as a non-offending partner (Kamitz & Gannon, 2024b), reported being judged or fearing judgement for their relationship decision.

This ties in with the literature suggesting that the courtesy stigma affecting non-offending partners may arise specifically from their refusal to sever ties with their partner who has offended even though they are in the “privileged” position of not being considered genetically contaminated (Condry, 2011). Specifically, the current research posits that non-offending partners may be judged to be, at best, stupid or naïve for not leaving their partner, given the potential danger their partners who have offended are perceived to pose. At worst, they are viewed as bad parents who put their needs before those of their children. The stereotypical construction of the non-offending partner as the mother of an intrafamilial abuse victim who is aware of and even permits abuse for the sake of her romantic relationship, and thus is responsible for the abuse, seems to be reflected here (e.g., Azzopardi et al., 2018; Pretorius et al., 2011).

The second construct assessed by then APPSO was participants’ Behavioral Intent toward non-offending partners (e.g., “I would be understanding toward a non-offending partner of someone who has committed a sexual offense”). This explicitly measured how participants may approach and treat non-offending partners in interpersonal interactions. Additionally, a third separate construct measured participants’ Judgement of Non-Offending Partners’ Character which, in line with previous research (Plogher et al., 2016) contained items describing non-offending partners as weak, mentally ill, and deviant because of their association with someone who has sexually offended, irrespective of their relationship decision (e.g., “Non-offending partners of people who have sexually offended have a lower IQ than other people”). One finding of note here was that two items (items 21 & 22) consistently cross-loaded significantly onto both Judgement of Character and Behavioral Intent. These items may explain some of the reasons underlying participants’ Behavioral Intent toward non-offending partners as arising from a Judgement of their Character. Specifically, the two items assessed poor character as being primarily based on immorality (e.g., “Non-offending partners of individuals who have sexually offended are immoral”). Participants may be especially inclined to want to socially distance themselves (as measured by some items of Behavioral Intent) from those that they see as immoral and deviant. In contrast, the need for social distance as a means of avoiding courtesy stigma (e.g., Condry, 2011) may not be as linked to perceiving non-offending partners as vulnerable or weak (e.g., “Non-offending partners of those who have committed sexual offenses are timid and afraid”).

Lastly, a final construct assessed by the scale was how much Shame participants attributed to others’ identity as a non-offending partner (e.g., “Non-offending partners of those who have sexually offended should feel ashamed”). That is, to which degree participants endorsed the belief that non-offending partners should feel guilty or ashamed. While only made up of two items, this construct distinctly corresponds to non-offending partners’ reported perceptions of being shamed for an offense that they themselves did not commit (Kamitz & Gannon, 2024a). Such shaming may be both external, as evidenced by the scale developed in the current study, but may also be internalized, as shown by participants in previous research who reported feeling guilty for their partners’ offending (Kamitz & Gannon, 2024a). The shaming of non-offending partners may also be linked to a contamination by causal responsibility as hypothesized by previous research (Armitage et al., 2024), whereby non-offending partners are blamed and held responsible for the offense committed by their partner.

Study 3 assessed potential differences in attitudes toward non-offending partners between those who regularly encounter this population in their professional lives (i.e., police officers and social workers), and a general population sample. We had hypothesized that professionals would hold more negative attitudes than the general population, given that non-offending partners in previous research often emphasized their negative experiences with various professional agencies (Armitage et al., 2024; Duncan et al., 2022; Kamitz & Gannon, 2024a). However, we instead found that professionals working with non-offending partners expressed more positive attitudes toward non-offending partners as evidenced by significantly lower scores on the overall measure, each subscale/factor, and almost all items. One possible explanation for this discrepancy between our hypothesis and findings may be that, any judgement non-offending perceive from professionals, while not more negative than judgement received from members of the general population, may be more salient due to the importance of intervening agencies in influencing the non-offending partner’s situation. While social support from family and friends is doubtlessly important, police officers, social workers, and other professionals affiliated with the criminal justice system hold the power to make important decisions affecting the non-offending partner’s life. For example, they may decide over child protection plans which could outline that the person who has committed an offense is not permitted to remain in the same household as the non-offending partner and their children. As a result, non-offending partners likely subject professionals to a very high level of scrutiny. Nevertheless, our findings additionally mirror a long-standing line of research showing that those working with people who have sexually offended have more positive attitudes toward them than community samples (for a review, see Willis et al., 2010). One potential explanation for this may be that, in line with the contact hypothesis (Allport, 1954), the contact between non-offending partners and professionals could lead to reduced prejudice. Such a decrease in prejudice between two groups has been suggested to arise from the increased knowledge about and empathy toward the outgroup and decreased anxiety or fear of interacting with the outgroup that may result from intergroup contact (Allport, 1954). Some evidence for this already exists for those working with individuals who have sexually offended as professionals’ positive attitudes (when compared to the general population) were mediated by their knowledge about sexual abuse (Sanghara & Wilson, 2006).

The potential importance of contact in improving attitudes toward non-offending partners could be harnessed to actively reduce prejudice against them. The positive impact of contact on prejudice reductions is not only a robust social-psychological effect, but contact interventions based on this theory have also generally been shown to be effective at reducing prejudice (for a meta-analysis, see Clochard, 2022). Specifically, the affective components believed to be responsible for prejudice reduction—reduced anxiety and increased empathy—have been found to be major mediators for this effect (for a meta-analysis, see Pettigrew et al., 2011). Specifically, the importance of empathy does not only describe why police officers and social workers may hold more positive attitudes toward non-offending partners, but it also suggests that it may be crucial to educate those close to non-offending partners about the emotional difficulty of their situation. Given that previous literature has shown a positive effect of contact on the perceptions of those who have committed sexual offenses (Wurtele, 2021), future research may investigate the efficacy of contact-based interventions with non-offending partners.

Study 4, which assessed the APPSO’s construct and criterion-related validity, found that negative attitudes toward non-offending partners were related to a range of personal characteristics and also predictive of a greater intent to discriminate against them across a range of different scenarios. Here, it is particularly noteworthy that negative attitudes toward individuals who have sexually offended were strongly positively correlated with APPSO scores, indicating that negative attitudes toward those who have sexually offended and their non-offending partners and ex-partners go hand-in-hand. While courtesy stigma, as previously discussed had long been suggested to be at the root of the discriminate non-offending partners face, our research is the first to demonstrate these concepts to be linked. This study additionally demonstrated that more negative attitudes toward non-offending partners were more predictive of certain acts of discrimination than others. For instance, in line with Plogher et al. (2016) previous work, participants demonstrated lower willingness to hire non-offending partners for childcare compared to factory work, a decision which we showed was predicted significantly by discriminatory attitudes toward them. This finding suggests that the stigma and discrimination non-offending partners endure may be particularly strong and damaging as it pertains to interpersonal relationships.

Study 5 aimed to examine the ecological validity of the APPSO through an adequately powered laboratory-based hot sauce paradigm to measure behavioral aggression. Although no significant relationship was found between APPSO scores and behavioral aggression toward non-offending partners, the findings are nevertheless insightful as they suggest that discrimination against non-offending partners may manifest in more subtle, non-violent ways. This could include social exclusion in areas such as housing and employment, as shown in Study 4. Together with Study 4, these results suggest that while attitudes toward non-offending partners may not always translate into overt aggression, they can still lead to significant social and economic disadvantages for non-offending partners.

### Practical Implications

In addition to the theoretical implications of the current research, it also has important practical implications. The factorial structure and items of the scale provide much-needed insight into the different components of attitudes toward non-offending partners, some of which could be especially impactful for those in contact with this population. For instance, this study has supported the previously posed hypothesis that non-offending partners may be perceived as naïve or even stupid (Plogher et al., 2016), either inherently (Judgement of Character) or because of their decision to stay with their partner (Judgement of Relationship Decision), which would inevitably influence their treatment by professionals and the general population. Additionally, non-offending partners are commonly judged to be bad parents or even permissive of child abuse, as measured by several items in this scale and shown by previous research (e.g., Azzopardi et al., 2018; Pretorius et al., 2011). Despite this, non-offending partners are often used to safeguard their children, while their own needs are neglected (e.g., Bolen, 2003). The simultaneous construction of non-offending partners as bad parents and safeguards presents a contradiction that, because of its inherent importance to social work, should be explored further in both research and practice. Additionally, any practitioner aiming to incorporate non-offending partners into their partner’s rehabilitation process should reflect upon their own attitudes and possible judgements in order to work ethically with non-offending partners.

In addition to this, our research demonstrates that attitudes toward non-offending partners are highly predictive of discriminating against them, possibly largely due to their association with someone who has committed a sexual offense. This discrimination may often be subtle rather than overtly aggressive and so it is crucial for professionals working with this population not to dismiss the stigma that non-offending partners face as it may nevertheless have very serious consequences for them. These findings further highlight the need for interventions that focus on reducing discrimination and improving understanding of non-offending partners’ situations, particularly when it comes to more subtle or indirect harms.

If future research, as previously suggested, investigates and finds support for a link between increased contact with non-offending partners and more positive attitudes toward them, then contact could be employed to reduce such stigmatization of non-offending partners in professionals likely to encounter them. Such contact could be built into training programs for those working within Criminal Justice System-adjacent organizations or counselling services. While the contact hypothesis facilitators first proposed by Allport (1954)—common goals, equal status, no intergroup competition, and authority sanction—have not been found to be strictly necessary for intergroup contact to have a positive impact (Pettigrew et al., 2011), they could nonetheless contribute to its success. In this specific case, equal status and achieving common goals particularly could be focal points of guidelines for working with non-offending partners. Perceived equal status may especially be hindered if non-offending partners are perceived to be vulnerable or stupid, as previously discussed. Therefore, these stereotypes of non-offending partners as a homogeneous, naïve group should specifically be addressed and challenged in training programs and guidelines with all agencies. The measure developed in this study could be used to assess attitudes toward non-offending partners at baseline and subsequently after the completion of a contact-based training program to evaluate the training’s efficacy. It is crucial that any content is carefully considered first to ensure that negative stereotypes are not made more salient (for a review, see Willis et al., 2010).

In addition to targeted training programs, given the potentially influential impact of media portrayals of non-offending partners on the general public’s attitudes toward them, as discussed above, researchers and practitioners alike may wish to be more proactive to pre-empt further stigmatization of and negative attitudes toward non-offending partners as perpetuated by the media. For instance, academics and those working with non-offending partners may show a greater engagement with the media to correct such narratives, as recommended by Willis et al. (2010) concerning those with sexual offense histories. While approaching the topic with consideration and empathy for those who have been victimized, this may provide a more nuanced view of non-offending partners’ situations than the media currently provides.

### Limitations and Future Research Directions

First, the ambiguity of grouping non-offending partners of all of those who have committed a sexual offense may present an issue. As sexual offenses are a broad category encompassing a variety of different offense types with varying degrees of severity and impact on the victims, those completing the measure may have found it difficult to “settle” on one answer per item which reflects their attitudes toward all individuals in this heterogeneous group. Future research may wish to ask participants after completion of the measure whether they imagined the non-offending partners’ partners as having committed a specific type of offense. Additionally, it may also be investigated whether different offense types elicit different attitudes and responses toward non-offending partners by swapping “those who have sexually offended” with “those who have committed a/an [rape/sexual assault/act of voyeurism…].”

Second, rather than being the result of increased contact, as suggested by the contact hypothesis (Allport, 1954), more positive attitudes toward non-offending partners observed in police officers and social workers within Study 3 could also be a sign of social desirability. While participants were ensured of their anonymity, which has been previously shown to reduce response biases caused by social anxiety and social desirability (Joinson, 1999), emails for participation in this study were sent to prospective participants’ work email addresses and the scale directly assessed their attitudes toward a population important to their work. Thus, these participants may have been motivated to respond with exaggerated positive attitudes reflective of the attitudes they believe they should have as police officers and social workers.

The general public and individuals in prison can behave aggressively toward those believed to have sexually offended (Allison, 2000; Halpert, 2023). Consequently, Study 5 aimed to explore the relationship between attitudes toward non-offending partners and behavioral aggression toward them in a more ecologically valid context. However, no significant relationship was found between participants’ APPSO scores and aggressive behavior toward a non-offending partner. While this may be indicative of some unavoidable limitations of the hot sauce experimental design (i.e., being unable to measure all possible mediating motivations for not administering the hot sauce; Ritter & Eslea, 2005), it may also suggest that negative attitudes toward non-offending partners lead to discriminatory behavior rather than overtly aggressive behavior. Nevertheless, as shown in Study 4, more subtle discrimination may still be highly damaging and impact non-offending partners in crucial areas such as housing, income, and social standing. Future research should investigate what other forms of harm or discrimination may be faced by non-offending partners and how the APPSO may predict this. The hot sauce paradigm, while useful for exploring aggression in a laboratory setting, may not fully capture the negative behaviors usually encountered by non-offending partners specifically.

Nevertheless, the results of these five studies, together, indicate that the APPSO is a robust measure exhibiting decent psychometric properties across the general community as well as professionals who work with non-offending partners. Consequently, we believe that the APPSO is likely to be an important measure for furthering our understanding of attitudes toward non-offending partners and how these might be improved.

## Data Availability

The data and materials used in this study, along with a pre-registration and the supplementary materials, are available on the Open Science Framework at https://osf.io/9jw47/?view_only=e1f1aa7e30d3446cac83dea1cabd4244.

## Footnotes

1 See supplementary materials for eigenvalues and explained variance.

2 See supplementary materials for unstandardized regression weights, standard errors, significance values, and confidence intervals for the model.

3 Participants’ genders were not recorded due to a survey error.

4 See supplementary materials for descriptive statistics.

5 All assumptions for conducting correlation analyses were met. A sensitivity analysis showed that, with 80% power and at *α* = .05, the minimum effect size detectable was small to medium (*r*/*ρ* =  ± .15).

6 All assumptions for conducting t-tests were met. A sensitivity analysis showed that, with 80% power and at *α* = .05, the minimum effect size detectable was small to medium (*ρ* = .15).

7 All assumptions for conducting ANOVAs were met. A sensitivity analysis showed that, with 80% power and at *α* = .05, the minimum effect size detectable was small to medium (*f* = .17).

8 See supplementary materials for descriptive and correlation statistics between APPSO total and subscales and each of the vignette scenarios, as well as full regression statistics including for analyses predicting intent to discriminate from APPSO subscales.

9 The overall scale (*α* = .94) and each of the subscales—Judgement of Relationship Decision (*α* = .91), Behavioral Intent (*α* = .85), Judgement of Character (*α* = .84), and Shame (*α* = .85)—demonstrated “excellent” internal consistency.
